# Anæsthetics in Labor

**Published:** 1891-03

**Authors:** 


### ANÆSTHETICS IN LABOR.

(Proceedings of I. H. A., Wednesday, June 25th, 1890. Afternoon Session.)

Dr. Alice B. Campbell said—Everything should be done in the name of humanity to relieve the pangs of women in labor, and I should like to know if a homœopathic physician can consistently use an anæsthetic in normal labor. It is on this question that I should like to get the sense of this meeting.

Dr. Wesselhœft—I believe that in the great majority of cases of normal labor we get along better without any anæsthetic whatever. We all know, also, that in many abnormalities we can aid labor very materially with a well-selected homœopathic remedy.

Still there are certain occasional cases, especially in primiparæ, where, after the second stage of labor is nearly ended, the head presents at the vulva with the most agonizing suffering, and either we fail in our selection of the remedy, or it fails to act for mechanical reasons, where I say it is only merciful to give a few whiffs of Ether or Chloroform while the head is passing to alleviate this most agonizing and terrific suffering. A very few whiffs of Ether or Chloroform will be sufficient, and I do not at all believe in any more than enough to just obtund keen pain without approaching anywhere near complete narcosis.

I believe, too, that it is perfectly justifiable for us to use an anæsthetic in obstetrical operations. I do not think I will ever apply the forceps again without at least partial etherization. Many women, now, seem to expect Ether from the beginning of the pains to the end of the labor, and I think that that is all wrong. Very many cases get along better without a drop of an anæsthetic, but in many others it is, if not necessary, at least merciful to use it to the limited extent that I have indicated.

Dr. Stow—I should like to call attention to a probable effect of the administration of Ether at the last stage of labor. We all know that there is extreme distention and tension of the perineal muscles during the last throes of labor, and a little prior to the last; is it not highly probable that a little of an anæsthetic administered at that time, will relax those muscles and do much to prevent rupture and laceration?

If I have a case of dislocation or fracture, I would not attempt to use my own slight muscular strength against the contracted and rigid muscles of the patient. I should administer an anæsthetic and then proceed to the easy and safe reduction of the dislocation or fracture aided by the relaxation of the muscles produced by the anæsthesia. The same applies to the management of difficult cases of labor, and I think we should be left to our own judgment and to exigencies of the case in this matter. I have had cases where I gave anæsthetics for the express purpose of producing relaxation.

Dr. Kent—In these painful and extraordinary cases, as in all others, the first duty of the physician is to act for the best interests of his patients; he cannot always do this by being merely merciful. If, with the idea of saving pain, you use Chloroform early in a labor case, the symptoms which call for a remedy will be entirely obliterated. I sit by the side of the bed, watching and waiting for a symptom to arise upon which I can base a prescription which will relieve the suffering and prevent puerperal fever.

It seems merciful to relieve this great suffering promptly with Chloroform, but it is more merciful to relieve it in the only right way by the homœopathic remedy when this is possible, because the relief is a real one and beneficial in its effect on the whole case, instead of merely palliating the pain.

I indorse what Dr. Wesselhœft has said, but I do not think with Dr. Stow that we are justified in producing so deep a narcosis as to relax the muscles of the perineum. It would take a great deal of Ether to do that, and I believe in a few whiffs only.

Dr. Dever—As I look at it, labor is a natural physiological process, and, in a healthy woman, should be gone through without any drugs or medicines at all. If the process is in any way abnormal, then the woman is sick and needs the homœopathic remedy, which will relieve, as we all know, more quickly and more permanently than any anæsthetic. It must be very seldom, if ever, necessary to use anything else.

Dr. J. B. Bell—If enough Ether is given to relax the perineum, then the labor is going to stop, and so deep an anæsthesia is very apt to injure the child. Another point to consider is that post-partem hemorrhage is likely to follow.

Dr. J. V. Allen—I would like to ask Dr. Bell if he is sure that Ether or Chloroform have ordinarily any effect upon the involuntary muscles.

Dr. Bell—Yes, sir, I think they do.

Dr. Stow—I do not wish to go on record as recommending either Chloroform or Ether indiscriminately. I am not in the habit of so using them, but I have had cases where the administration of anæsthetics has very materially relieved suffering. I know that I have, without deep narcosis, produced sufficient relaxation of the muscular tissue to very materially aid the passage of the head and to prevent laceration of the perineal structures. I must differ from Dr. Bell when he says that anæsthetics affect the involuntary muscles. The heart continues to beat, the lungs to move, and the expelling power of the uterus is scarcely impaired under an anæsthetic. If you unwisely carry the effect so deep as to affect the involuntary muscles you kill the patient.

Dr. Hawley had never used Chloroform but twice and one of these occasions was a case of hour-glass contraction.

Dr. Fincke—This discussion is not necessary and does not answer the question that Dr. Campbell asked. She asked whether it was, in the opinion of this Society, legitimate for a homœopath to give anæsthetics in normal labors in a sentimental way to stop pain. I say that if everything goes well she should bear some pain, for the woman will be loved better and will love her children better if she suffers some pain.

Pain is a part of labor, and if everything is normal we should allow nature to do her own work.

I have seen many cases go wrong because the doctor had no time to properly attend the case, so hurried matters up, to the harm both of the mother and child. Many women that have a quite natural child-birth will cry out with pain and say that they are going to die. If you give them a tiny pellet of Aconite it all passes off and the child will be born all right.

Dr. Carr—When I first began practice I was impressed with such great sympathy for my patients that I administered Chloroform in every case. But I did not know as much about Homœopathy at that time as I do now. It was not until I had some very untoward results that I turned my attention to the remedies. Aconite, Kali-carb., and Chamomilla have served me well in such cases.

Dr. H. C. Allen—One point has been overlooked in this discussion. One reason why we should not give anæsthetics in labor is because the old school do. The farther we keep away from their methods the better for all concerned. A woman is more susceptible during labor and pregnancy to the action of remedies than at any other time. Moreover, the symptoms of the mother corrected during gestation, and just prior to confinement, tend to make the labor normal. An anæsthetic masks symptoms, prolongs suffering in the end, increases the liability to hemorrhage, to mastitis, and other troubles of the mammary gland. The closer we keep to the dynamis of the remedy the better for the mother and the better for the child. Keep to the indicated remedy; it does as much for both mother and child during labor as it does during the dynamis of gestation. Anæsthetics destroy the indications for the remedies and increase the danger of hemorrhage.

Dr. Wesselhœft—I do not want to be understood, nor do I want it to go on record that I advise the use of anæsthetics, except in certain rare cases such as I have mentioned. As for our allopathic friends using or not using anæsthetics, I do not think that has anything to do with it. They are abandoning the practice more and more. I think we should use some Chloroform whenever the forceps have to be applied; also in some cases, especially primiparæ, in the last moments when the child's head is bursting through the vulva, and the woman is enduring the most excruciating tortures. Just a few whiffs are enough and it is all over. I have never seen bad results from it, and I have had women thank me for those few whiffs. Mind, that in the great majority of cases, I say we do *not* need it and are better off without, but in the cases I speak of I am glad to give relief by its use.

Dr. Alice B. Campbell—I am glad to have gotten these expressions of opinion. In my estimation those few whiffs are going to lower Dr. Wesselhœft a little. I am very jealous of the reputation of this Society, and my gratification is great to hear Dr. H. C. Allen and others stand up for true Homœopathy.

I believe Dr. Wesselhœft thinks he is right, but I do not.

Whether it is Dr. Stow with his complete narcosis, or Dr. Wesselhœft with his few whiffs, the principle is the same. If you do the same as the allopaths, wherein lies the difference? Can we not stand alone? Must we depend upon their miserable expedients? I wish the homœopathic use of Chloroform in normal labor were wiped out of existence. I have followed where it has been used and have always found more or less trouble generally in connection with lactation.

Dr. Kimball—Where the final pains are of so excruciating a character, can the labor be called normal?

Dr. Guernsey—I was called to a case in which a girl about seventeen in labor was in the most horrible convulsions. The immediate use of Ether relieved the pains, and I do not think five minutes elapsed before the child was born.

Dr. J. B. Bell—We must concede a little here I think. If things were just as we should like them to be, we would have painless labors, and we would have no surgery. I think that Ether may be safely and comfortably used toward the close of labor, when, according to the judgment of the physician, it is best.

Dr. Wesselhœft—For Heaven's sake, do not understand me to advise the use of Ether right along, from the moment the woman begins to cry out. Many of the women are abnormal nowadays, and can hardly have a normal labor. When we have a mechanical tumor pressing against and distending to the point of rupture, the vulva, with that horrible agony depicted on the face of the woman, I have used a little Chloroform, and I am glad I did.

Dr. Hoyne—I have found that women nowadays are educated up to Chloroform, and will not have a doctor attend them in confinement unless he will give them Chloroform. Very little is necessary, and only toward the close of labor. I have never heard of its doing any damage when used in that way.

Dr. Fincke—I wish Dr. Wesselhœft had tried the remedy just at that point (perhaps, Aconite would have done it), because we would have learned something. Suppose, in cases similar to this, we try the remedies, and then we will know. How do we know that the anæsthetics do not have an after effect? There must be some reaction, I think, but I do not know enough about it to say, and should like to hear from somebody who knows.

I know of a young girl upon whom laughing gas, administered to have a tooth drawn, produced very serious effects; also a widow who was under the relaxation of anæsthesia for many hours. In course of time she began to weep, and she wept herself to death. I do not know whether this can be due to the anæsthetic or not. It is only possible, and would have to be proved.

Dr. Dever—These desperate cases are the very ones that need homœopathic treatment, and the very ones in which our remedies will do good.

Dr. Thomson—I have never used an anæsthetic in a normal labor. The more excruciating the pain, the quicker will the indicated remedy ease it. It is just as criminal to take away that pain with an anæsthetic, as it would be to cut down the red flag, the danger signal, and let two trains come together. The pains are a part of and coincident with the contractions of the uterus, and we should not interfere with them.

Dr. H. C. Allen—Let us settle this question by observing for the next three years all the obstetrical cases in which Chloroform or Ether has been given, and see how they get on. Observe especially the progress of lactation. My personal experience is that troubles in lactation are very common when an anæsthetic has been used; either caked breast or suppression of milk or some similar trouble.

Dr. W. J. Guernsey—What is normal labor? Is such a case as Dr. Wesselhœft mentioned normal? I think not.

Dr. Wesselhœft—As I said before, we have abnormal women to deal with. A normal woman who uses her muscles, who has strength and ability, who has never injured herself by wrong dressing will have a normal baby by means of a normal labor. Such a woman will not present a head twelve inches long bursting through the vulva with such horrible pains.

I am not talking about painful contractions of the uterus at all; these are the normal pains of labor. I am speaking of the last pains due to the mechanical bursting, tearing of the vulva by an abnormal head. No remedy could correct that. It is not a dynamic condition, and a few whiffs of an anæsthetic will do the whole business. It can do no more harm than a few whiffs of Amyl-nitrite.

Dr. J. V. Allen—I have just one case in which I used Ether. At the end of the first stage of labor I wanted to use the forceps, but the patient would not allow me. I had been there three or four days and the child was ready to be expelled. I got some Ether, applied a towel to her nose, and in a few minutes the labor was over.

Dr. Custis—The best argument against the anæsthetic yet advanced is Dr. Kent's—that we thereby cover up symptoms and cannot make a prescription. The drift of the discussion amounts to this: never give an anæsthetic in normal labor. In abnormal labor correct the condition with the indicated remedy if you can, and if you cannot, help the woman with the anæsthetic.

Dr. Alice B. Campbell—I wish to sustain Dr. Wesselhœft in his position that many of the women of to-day are abnormal, and we are likely to have them remain so if we obey all their prejudices and whims, as Dr. Hoyne would have us do.

Dr. Hitchcock—If the women themselves are abnormal, are we justified in using abnormal measures in treating them?

Dr. Kent—Suppose your woman is under the influence of an anæsthetic and an active hemorrhage comes on, what are you going to do for her, with her symptoms masked by that benumbing influence?

Dr. Wesselhœft—I have had just such a case. The woman was under an anæsthetic. The child was delivered, when a sudden post-partum hemorrhage came on, such as I had never seen before. The doctor who was connected with me in the case, ran for his instruments. I ran for my Ipecac and gave it. It stopped immediately. It was one straight, hot gush of bright, red blood as thick as a man's arm. I knew that I would have to work quickly; I have Ipecac always near me in labor cases.

Dr. Kent—Then every case of hemorrhage coming on under an anæsthetic must call for Ipecac.

Dr. Brownell—It seems to me that the Ipecac does not deserve any credit for stopping that hemorrhage. The sudden contraction of the uterus that forced out that sudden gush of blood also stopped it.

Dr. Rushmore—I recall at this moment three cases of labor in which I was called in consultation. They were all instrumental cases and in each case an anæsthetic was used.

In two of the three the child was past resuscitation and in the third resuscitation was very difficult.

